# Analysis of Anakinra Therapy for the Deficiency of Interleukin-1 Receptor Antagonist through Clinical Evidence

**DOI:** 10.3390/jcm13041026

**Published:** 2024-02-10

**Authors:** Kathryn Pillai, Joshua Pillai, Jun Ling

**Affiliations:** 1 Department of Medical Education, School of Medicine, California University of Science and Medicine, 1501 Violet St, Colton, CA 92324, USA; 2 Department of Biological Sciences, Irvine Virtual Academy Secondary, 3387 Barranca Pkwy, Irvine, CA 92606, USA

**Keywords:** autoinflammatory diseases, deficiency of interluekin-1 receptor antagonist (DIRA), anakinra, IL-1RA, IL-1RN

## Abstract

Background. Deficiency of interleukin-1 receptor antagonist (DIRA) is a rare life-threatening autosomal recessive autoinflammatory disease with symptoms including but not limited to osteomyelitis, periostitis, and systemic inflammation. DIRA is developed from the loss-of-function biallelic mutations of the IL1RN gene that encodes IL-1 receptor antagonist (IL-1RA), leading to the unchecked pro-inflammatory signaling and subsequent systemic inflammation. Thus, anakinra as the recombinant IL-1RA has become the primary drug to treat DIRA. Although anakinra has been effective for the complete remission of DIRA, it has also shown various side effects. To confirm the efficacy and safety issues associated with DIRA treatment, we conducted a literature review and secondary data analysis to enhance our understanding on this important topic. Methods. Through comprehensive literature search, we have identified 15 papers with 25 patients studied. The demographic, clinical, and genetic data were extracted, followed by statistical analysis to support the physiological mechanisms of anakinra treatment. Results. Through the literature review and data analysis, it was found that 88% of patients had complete clinical remission of DIRA upon continual treatment with anakinra; patients had a mean improvement of Hemoglobin (+3.18 g/dL), Erythrocyte Sedimentation Rate (−53.4 mm/h), and C-reactive Protein (−135.45 mg/L) levels, suggesting that the improvement of hematopoietic function and inflammation is a mechanism for anakinra treatment. Various genetic variants were also identified from the patient data that cause the loss of function of IL-1RA, providing real patient genomic data to support the anakinra treatment. Conclusions. Considering the inconsistency and certain variations from clinical research influenced by specific conditions, this review along with the data analysis confirms the efficacy and safety of anakinra treatment for DIRA.

## 1. Introduction

The interleukin-1 (IL-1) family is a group of 11 cytokines and 10 receptors that regulate inflammatory responses with various roles. Some of them, such as IL-1α, IL-33, IL-36, and IL-1β, mediate a pro-inflammatory response [1], whereas others like IL-37 and IL-38 are anti-inflammatory [1]. When the pro-inflammatory cytokines of the IL-1 family bind to the cognate receptor IL-1R1, they recruit the secondary receptor IL-1RacP [2], thus initiating IL-1 signaling through two mediators, myeloid differentiation primary response 88 (MyD88) and interleukin-1 receptor associated kinases 4 (IRAK 4), to eventually activate nuclear factor κB (NFκB) and activator protein 1 (AP1). Both transcription factors will regulate target gene expression to mediate IL-1 functions. Based on the given signal transduction pathways, tight regulation is required to prevent detrimental effects that could lead to chronic inflammatory disorders.

Among the IL-1 family, IL-1α plays pleiotropic roles in both inflammation and hematopoiesis; it is widely expressed in various cells including epithelial, endothelial, stromal cells, neutrophils, and activated macrophages [3]. Under the condition of cellular damage, necrotic cells release IL-1α into the extracellular environment as an alarmin [4]. In addition, IL-1α is also induced in hematopoietic and non-hematopoietic cells in response to inflammatory stimuli. IL-1RA exists as an antagonist to compete with IL-1 for binding to IL-1R1. The balance between IL-1RA and IL-1 becomes very crucial for the development of a variety of inflammatory diseases such as Deficiency of IL-1 Receptor Antagonist (DIRA), Rheumatoid Arthritis (RA), Gastric Cancer, and Osteoporosis.

DIRA is a rare autoinflammatory disorder characterized by marked skin and bone involvement, and the elevation of acute phase reactants [5]. DIRA is caused by a loss-of-function biallelic mutation occurring in the IL1RN gene that prevents the expression of active IL-1RA, causing unchecked pro-inflammatory signaling and subsequent systemic inflammation [6]. DIRA has a challenging feature with very early onset. As early as the first week of life, DIRA manifesting symptoms can be developed, including pustular rash, the widening of ribs, periosteal reaction, multifocal osteolytic reactions, cervical vertebral fusion, hepatosplenomegaly, and multifocal osteomyelitis [7]. The primary drug choice for DIRA is anakinra.

Anakinra is a recombinant form of the human IL-1RA protein. The primary difference between the recombinant and human form is that the recombinant contains an additional methionine residue in the amino terminus [7]. Anakinra was approved by the U.S. Food and Drug Administration (FDA) in December 2020 for the treatment of DIRA. Currently, anakinra is the primary anti-IL1 therapeutic due to its short half-life, safety records, and subcutaneous route of delivery.

Although anakinra has been approved by the FDA for DIRA therapy, there is no previous review to evaluate anakinra’s clinical efficacy and safety as a recombinant analog of IL-1RA in the treatment of this autoinflammatory disease. Here, through a literature review and an analysis of the data from selected clinical case reports, we identified that anakinra decreased acute phase reactants levels and improved anemia in patients after the treatment with anakinra, supporting the efficacy of anakinra treatment. The safety of this treatment was also confirmed.

## 2. Materials and Methods

### 2.1. Literature Search Strategy

A literature search was conducted through PubMed, EMBASE, and Google Scholar, including articles from inception: keywords “DIRA” or “Deficiency of IL-1RA” and “Anakinra” were used to filter the studies evaluating the efficacy of anakinra in DIRA therapy. The language was restricted to English only.

**Figure 1:**
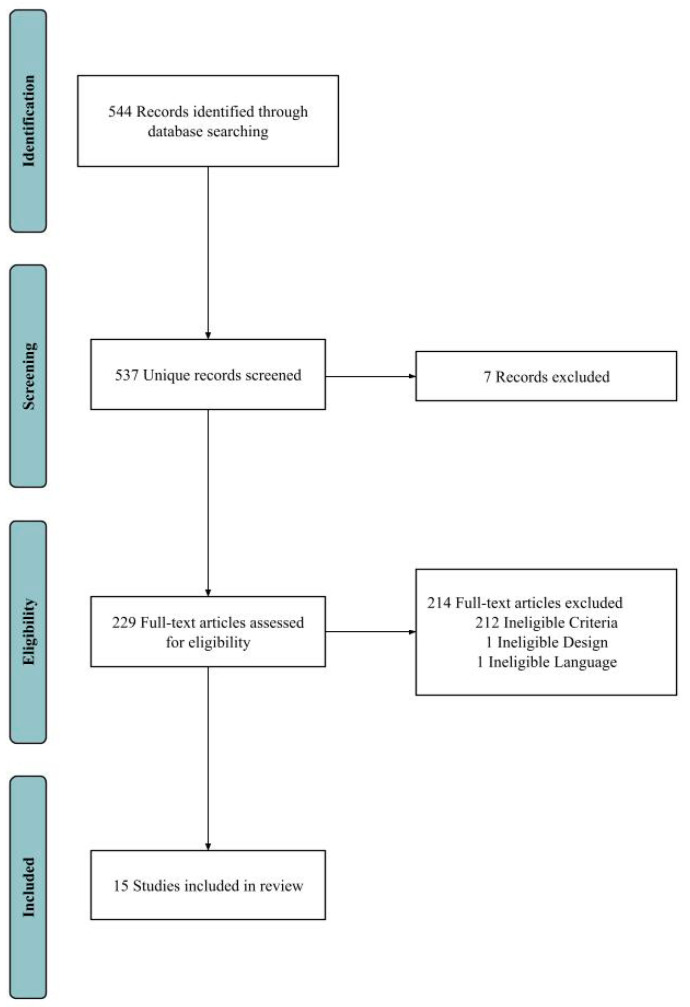
Literature search and screening process for this review. A total of 544 studies were retrieved from all databases. All studies were screened. Seven duplicate articles were identified and removed. Full-text articles were assessed for eligibility and 214 studies were removed due to ineligible language, design, or criteria. In total, 15 studies were finally selected for this review.

Gray literature was not included in the literature search. We included clinical trials, clinical studies, case reports, comparative studies, original articles, meta-analysis, observational studies, and twin studies in the literature search. The detailed literature search and screening process is depicted below (Figure 1).

### 2.2. Study Selection and Eligibility Criteria

Two paired reviewers independently conducted a title and abstract screening. We assessed the abstracts to identify articles that discussed anakinra as the main treatment for DIRA and removed duplicate articles. Abstracts were also assessed to see if the efficacy of anakinra was discussed with laboratory values or other means. Side effects of anakinra were also considered. Abstracts that discussed anakinra for the treatment of other rheumatological diseases but not DIRA were excluded from this study. Following screening, both reviewers independently evaluated the full text of papers for eligibility. For laboratory results, 10 patients were excluded for Hemoglobin (HB), 7 patients for Erythrocyte Sedimentation Rate (ESR), and 8 patients for C-Reactive Protein (CRP) because either the data were missing or not fully reported pre- or post-treatment, respectively.

### 2.3. Data Extraction

The data were extracted from the selected papers [6,7,8,9,10,11,12,13,14,15,16,17,18,19,20] on the age of the presentation of DIRA symptoms, sex, region of origin, reported cytogenetic abnormalities, all previous treatments, consanguinity status, side effects from anakinra treatment, and the reported common clinical presentation. HB, CRP, and ESR were also extracted from these papers. These markers were included to comprehensively assess DIRA as an autoinflammatory disease. The efficacy of anakinra for the treatment of DIRA has been evaluated with the CRP and ESR laboratory values pre- and post-treatment. HB laboratory values have also reported pre- and post-treatment to evaluate anemia associated with chronic diseases.

### 2.4. Statistical Analysis

Statistical analysis in this work was performed using GraphPad Prism (version 9.5.1) with its embedded algorithms. The Wilcoxon rank-sum test was applied to evaluate the significance of laboratory results pre- and post-treatment.

## 3. Results

All patient demographics and medical history were extracted from 15 selected papers as shown in Table 1. Of the 15 studies included in this review, a total of 25 patients were treated with anakinra for DIRA [7,8,9,10,11,12,13,14,15,16,17,18,19,20,21]. In total, 19 patients (*n* = 19, 76%) were 4-weeks-old or younger, and a total of 23 patients (*n* = 23, 92%) were under 17-weeks-old (Table 1). The sex of all the patients was primarily male, making up 16 total (*n* = 16, 64%). The most common ethnicities of patients were Dutch (*n* = 6, 24%), Lebanese (*n* = 3, 12%), Puerto Rican (*n* = 4, 16%), and Brazilian (*n* = 3, 12%). A total of 13 patients (*n* = 13, 52%) were non-consanguineous and 9 patients (*n* = 9, 36%) were consanguineous.

The common clinical manifestations of DIRA were pustular rashes (*n* = 21, 84%), multifocal osteolytic lesions (*n* = 17, 68%), widening of ribs (*n* = 15, 60%), periosteal reaction (*n* = 13, 52%), and multifocal osteomyelitis (*n* = 12, 48%). However, Abdwani et al. [18] reported chronic diarrhea as the initial unusual presentation of DIRA, suggesting that inflammation might also disturb the gastrointestinal immunity leading to diarrhea as one of systemic symptoms of DIRA. Many patients also exhibited anemia. A small portion of patients also had extramedullary hematopoiesis leading to hepatomegaly and hepatosplenomegaly, all of which are consistent with a severe effect of DIRA on bone inflammation that might cause malfunction of hematopoiesis. All other clinical symptoms are listed in Table 1.

To evaluate the treatment efficacy of anakinra, we also looked into the details of the treatment regimens in each case. Before treatment with anakinra, 16 patients (*n* = 16, 64%) were previously treated with prednisolone/methylprednisolone; 17 patients (*n* = 17, 68%) with oral/intravenous antibiotics; Canakinumab, an IL-1β blocker, was also given to a few patients (*n* = 2, 8%). These various pre-treatments might decrease inflammation, control opportunistic infections, or play a complementary role for anakinra treatment, therefore favoring the outcome of anakinra treatment. Further clinical trials are needed to evaluate the effects of treatment with anakinra alone or in combination with other drugs.

### 3.1. Treatment Effects of Anakinra and the Underlying Genetic Mechanisms

To further understand the genetic mechanisms underlying the symptoms of DIRA patients and their relationship with responses to anakinra treatment, the genetic variants of IL1RN were extracted from those 15 studies whenever available. It was found that there were many types of mutations involved, including long and short region deletion, missense, nonsense, and frame-shift mutations, all of which led to the defects of IL-1RA expression and/or inactivation. Such diversity in genetic mutations also suggests the high variations in DIRA symptoms, including the types of symptoms, severity, and time of onset. As shown in Table 2, the most prevalent nucleotide variation in the IL1RN gene leading to DIRA was c.229G>T (*n* = 5, 20%), which led to a nonsense mutation on the amino acid sequence at position 77 (E77X). A 175-kb deletion mutation on chromosome 2q13 (*n* = 5, 20%) was equally prevalent; this variant is close to the IL1RN locus at 2q14.1, suggesting a high possibility of affecting IL1RN gene expression. These different variant frequencies may provide valuable biomarkers for the precision medicine diagnosis of DIRA.

### 3.2. Physiological Responses to Anakinra Treatment

**Figure 2:**
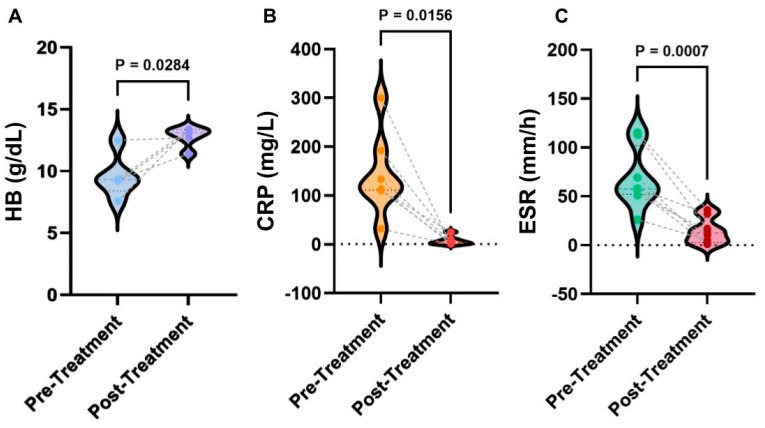
Statistical analyses of laboratory results of anakinra therapy. (**A**) Comparison of HB levels pre- and post-treatment. (**B**) Comparison of CRP levels pre- and post-treatment. (**C**) Comparison of ESR pre- and post-treatment. *p* values were determined from the Wilcoxon rank-sum test. The dataset for all laboratory data is reported in Supplemental Materials.

To understand the physiological mechanisms behind anakinra treatment, the laboratory testing data were further analyzed with three typical parameters summarized Figure 2. Overall, after the treatment of anakinra, HB levels significantly increased (*p* = 0.0284), CRP levels significantly lowered (*p* = 0.0156), and ESR levels significantly lowered (*p* = 0.0007). From the data analysis, it was found that before treatment, HB levels were 9.3 with an IQR (interquartile range) of 2.55 g/dL, CRP levels were 114 with an IQR of 81.8 mg/L, and ESR levels were 57.5 with an IQR of 37.5 mm/h, respectively. The HB levels after treatment were 13.1 with an IQR of 1.3 g/dL, CRP levels were 4.2 with an IQR of 9.5 mg/L, and ESR were 12.5 with an IQR of 21 mm/h. It was found that 88% of patients had complete clinical remission of DIRA upon continual treatment with anakinra; patients had a mean improvement of Hemoglobin (+3.18 g/dL), Erythrocyte Sedimentation Rate (−53.4 mm/h), and C-reactive Protein (−135.45 mg/L) levels. These results suggest that the improvement of hematopoietic function indicated by HB and ESR and inflammation indicated by CRP is among the major mechanisms for the action of anakinra. Further studies are needed to test broader factors of inflammation and immune responses, such as cytokines (e.g., IL-1α and β, INF-γ) and Th1 and Th2 activation, to advance our understanding. The datasets for these analyses were included in the Supplementary Materials (Tables S1–S4).

### 3.3. Anakinra Treatment Outcomes and Side Effects

After beginning treatment of anakinra, the median duration of patient follow-up was 10 days with an IQR of 9 days. The outlier was 30 days as reported in one study by Mendonça et al. [12]. The total amount of days of anakinra was a median of 182.5 days with an IQR of 213.75 days; no outliers were reported. There was only one reported relapse that occurred upon anakinra treatment [9], where the patient had injection site reactions and experienced elevated inflammatory markers. Additionally, there were no secondary relapse reported. There was only one primary failure caused by anakinra reported in Ziaee et al. [14], where the patient died due to respiratory distress caused by the administration of anakinra.

After the administration of anakinra, there was a clinical remission of DIRA. However, some patients experienced side effects as summarized in Table 3. In the case report by Mendonca et al. [8], the patient experienced an urticarial rash within fifteen minutes of administration of anakinra for the first time. This was well controlled by pre-medication with diphenhydramine, followed by the administration of anakinra. However, in a case report by Ziaee et al. [14], a patient showed signs of respiratory distress for two weeks because of the administration of anakinra. This patient also showed bone and skin complications. Then, anakinra was discontinued, etanercept and prednisolone were started, and an improvement was observed. Although there were these side effects, they can still be managed as evidenced by these clinical studies, providing a future direction to optimize anakinra treatment.

## 4. Discussion

The primary ethnicities of DIRA patients were Dutch, Puerto Rican, and Brazilian as listed in Table 1, and their heterozygous relatives were not identified to exhibit any symptoms of DIRA as shown in Table 2. These genetic variants are first generation homozygous mutations and have been characterized in isolated geographical regions. The allele frequencies of the founder mutations in Puerto Rico (Arecibo) and Newfoundland have been estimated by Reddy et al. [6] and Aksentijevich et al. [7] to be 1.3% and 0.2%, respectively. In such isolated geographic regions, genetic counseling and prenatal screening is recommended for identification.

Recent studies such as Bittles et al. [21] showed that consanguinity would increase the likelihood of complex diseases if rare autosomal recessive alleles were related. Given that DIRA is an autosomal recessive autoinflammatory condition, patients with consanguineous parents should have a higher prevalence than patients with non-consanguineous parents. However, in this study, as shown in Table 1, patients were primarily born to non-consanguineous parents. This could be potentially explained because of a higher population of non-consanguineous parents than those of consanguineous parents. Due to the limited sample size in this review, further studies are required to precisely determine the correlation of consanguinity with DIRA.

In multiple studies, such as Kuemmerle-Deschner et al. [9] and Minkis et al. [13], DIRA patients were first diagnosed with pustular psoriasis based on skin biopsy and clinical presentation. However, genome-wide sequencing identified mutations in the IL1RN gene and the diagnosis of DIRA. Across all studies, Sanger sequencing, SNP array, PCR, in silico modeling, and Next-Generation Sequencing were utilized to identify DIRA to accurately differentiate DIRA from other similar systemic autoinflammatory disorders, such as chronic nonbacterial osteomyelitis. Thus, molecular diagnosis with the following genes has been incorporated: IL1RN, MEFV, MVK, TNFRSF1A, NLRP12, NLRP3, NOD2, LPIN2, PSMB8, PSTPIP1, which provides a rapid and effective genetic screening for early treatment as the delayed treatment can cause major complications such as severe osteomyelitis.

The primary limitation of this study is the possibility of publication bias in all studies. From Nissen et al. [22], case reports with positive outcomes are favored in reporting over those with treatment failures. Therefore, there can be potential under estimation of adverse effects caused by anakinra therapy. Poorly documented observations of failed and successful treatments were excluded in this study.

Another limitation of this study is a lack of clinical trials. Given the nature of DIRA as an ultra-rare disorder, having clinical trials with enough participants would be very challenging. We suggest the development of an international registry of patients treated for DIRA to improve documentation of patient outcomes.

From this review, we obtained more evidence on the effectiveness of anakinra therapy for the treatment of DIRA. With a dose of 2–3 mg/kg/day anakinra, complete remission of DIRA is possible. Although most patients have reported no side effects of anakinra, some patients have reported adverse effects from anakinra. This could be attributed to the different genetic predisposition of each individual patient. With proper desensitization to anakinra with diphenhydramine, the improvement of treatment and full clinical remission of DIRA can be reached.

## 5. Conclusions

DIRA is a rare autoinflammatory disorder with early onset (1–8 weeks) and poor prognosis (systemic inflammation, mortality, etc.). Due to its similarity to other common autoinflammatory disorders, it has been difficult to diagnose DIRA. In this review, we evaluated the efficacy of anakinra in the treatment of DIRA and reported statistical analyses of laboratory data from the selected studies. The results indicated that most patients had no adverse effects to anakinra with a response rate of 96% (*n* = 24). In summary, our study confirms that complete remission of DIRA is possible through anakinra therapy. However, further studies with larger-scale collaborations and sample sizes are required to confirm the efficacy of anakinra for treating DIRA.

## Figures and Tables

**Table 1:** Ages, Sex, Ethnicity, Consanguinity, Clinical Presentation, and Treatments of DIRA Patients.

Median Age (Days)	IQR (Days)	Outlier (Days)
8	24.208	121.667, 121.667, 364 *
**Sex**	***n* = 25**	**Percentages (%)**
Male	16	64
Female	9	36
**Ethnicity**	***n* = 25**	**Percentages (%)**
Asian Indian	1	5
Brazilian	3	12
Puerto Rican	4	16
Turkish	1	4
Iranian-Persian	1	4
Lebanese	3	12
Portuguese and French	1	4
Canadian (Newfoundland)	1	4
Dutch	6	24
Not Known	3	12
**Consanguinity**	***n* = 25**	**Percentages (%)**
Non-consanguineous	13	52
Consanguineous	9	36
Not Known	3	12
**Clinical Presentation**	***n* = 25**	**Percentages (%)**
Pustular Rash	21	84
Widening of Ribs	15	60
Periosteal Reaction	13	52
Multifocal Osteolytic Lesions	17	68
Cervical Vertebral Fusion	4	16
Hepatosplenomegaly	6	24
Multifocal Osteomyelitis	12	48
Hepatomegaly	1	4
**Treatments**	***n* = 25**	**Percentages (%)**
Prednisone/Prednislone/Methylprednisolone	16	64
Canakinumab	2	8
IVIG	2	8
Soriatane (acitretin)	2	8
Etanercept	2	8
Bisphosphonates IV	1	4
Methotrexate	2	8
Azathioprine	1	4
Cyclosporine	2	8
Thalidomide	1	4
Interferon-γ	1	4
Oral/Intravenous Antibiotics	17	68
Antifungals	2	8

* The outlier ages were reported by Mendonça et al. [12], Sakran et al. [15], and Kuemmerle-Deschner et al. [9], respectively.

**Table 2:** Genetic variations in DIRA patients.

Genetic Variations	*n* = 25	Percentages (%)
homozygous 22,216 bp deletion	1	4
homozygous stop variant c.62C>G; p. Ser21*	1	4
c.396delC, stop codon c534 position	1	4
p.Asp72_Ile76del; p.Q45* (rs1019766125)	1	4
NM_001318914.2:c.54delC;p.Asn18Lysfs*4,	2	8
c.160C>T; (Q54X)	1	4
15-bp deletion, (c.213_227delAGATGTGGTACCCAT; p.Asp72_Ile76del)	2	8
175-kb deletion on chromosome 2q13	5	20
(NM_173841.2:c.364C>T:p.[Gln122Ter])	1	4
E77X and C140delC leading to p.T47TfsX4; heterozygous	1	4
(c.156_157delCA) leading to frameshift mutation N52KfsX25,	1	4
c.229G>T; resultant amino acid mutation, E77X	5	20
c.160C>T; resultant amino acid mutation, Q54X	2	8
Not known	1	4

**Table 3:** Known side effects of anakinra.

Side Effects of Anakinra	*n* = 25	Percentages (%)
Elevated Acute Phase Reactant	1 *	4
No Reported Effects	22	88
Respiratory Distress & Death	1 **	4
Urticarial Rash	1 ***	4

* Kuemmerle-Deschner et al. [9] reported this side effect after administration of anakinra. ** Ziaee et al. [14] reported these side effects after administration of anakinra. *** Mendonca et al. [8] reported this side effect after administration of anakinra.

## Data Availability

The data presented in this study are available in Supplementary Materials.

## Supplementary Materials

The following supporting information can be downloaded at: https://www.mdpi.com/article/10.3390/jcm13041026/s1. Table S1: Hemoglobin results from anakinra therapy; Table S2: Erythrocyte Sedimentation Rate results from anakinra therapy; Table S3: C-Reactive Protein results from anakinra therapy; Table S4: Efficacy of anakinra therapy in patients.
