# Supplementary material for: Analysis of Anakinra Therapy for the Deficiency of Interleukin-1 Receptor Antagonist through Clinical Evidence
Source: J Clin Med. 2024 Feb 10;13(4):1026. doi: 10.3390/jcm13041026 (PMC10888712; doi:10.3390/jcm13041026)
Supplement: Supplementary file 1 [file jcm-13-01026-s001.zip › jcm-2786636-supplementary.pdf]

SUPPLEMENTAL INFORMATION

Analysis for Anakinra therapy for the Deficiency of Interleukin-1 Receptor Antagonist through Clinical Evidence

Kathryn Pillai, Joshua Pillai, Jun Ling*

*Correspondence should be addressed to J.L. (jun.ling@cusm.edu)

This PDF includes:

Supplementary Table 1

Supplementary Table 2

Supplementary Table 3

Supplementary Table 4

Supplementary Table 1. Hemoglobin results from anakinra therapy (*n* = 5).

Pre-Treatment Hemoglobin (g/dL)	Post-Treatment Hemoglobin (g/dL)
9.3	11.4
12.5	12.7
7.6	13.4
9.4	13.3
9.2	13.1

Supplementary Table 2. Erythrocyte Sedimentation Rate results from anakinra therapy (*n* = 8).

Pre-Treatment Erythrocyte Sedimentation Rate (mm/h)	Post-Treatment Erythrocyte Sedimentation Rate (mm/h)
113	17
26	5
57	10
56	0.83
51	15
69	2
58	32
115	36

Supplementary Table 3. C-Reactive Protein results from anakinra therapy (*n* = 7).

Pre-Treatment C-Reactive Protein (mg/L)	Post-Treatment C-Reactive Protein (mg/L)
110.7	4.2
112	25
192.5	10
114	1.3
32	0.5
300	0
134	6.1

Supplementary Table 4. Efficacy of anakinra therapy in patients.

**Follow-up (median)**	**IQR (Outliers)**	**Sample Size (*n*)****
10	9 (30*)	10
**Time of onset (median)**	**IQR (Outliers)**	**Sample Size (*n*)****
182.5	213.75 (None)	10
**Relapse (*n*)**	**No Relapse (*n*)**	**Sample Size (*n*)**
1	24	25
**Primary Failure (*n*)**	**No Primary Failure (*n*)**	**Sample Size (*n*)**
1	24	25

*Mendonça et al. reported this outlier follow-up date. **15 patients did not have reported follow-up dates and time of onset reported in the case report.
